# Compensation of Rotary Encoders Using Fourier Expansion-Back Propagation Neural Network Optimized by Genetic Algorithm

**DOI:** 10.3390/s20092603

**Published:** 2020-05-03

**Authors:** Hua-Kun Jia, Lian-Dong Yu, Yi-Zhou Jiang, Hui-Ning Zhao, Jia-Ming Cao

**Affiliations:** School of Instrument Science and Opto-Electronics Engineering, Hefei University of Technology, Hefei 230009, China; huakun_jia@mail.hfut.edu.cn (H.-K.J.); jiangyizhou1@mail.hfut.edu.cn (Y.-Z.J.); hnzhao@mail.hfut.edu.cn (H.-N.Z.); cjmsaw1113@163.com (J.-M.C.)

**Keywords:** angle measurement error, BP neural network, genetic algorithm, rotary encoder, temperature compensation, instrument

## Abstract

The measurement accuracy of the precision instruments that contain rotation joints is influenced significantly by the rotary encoders that are installed in the rotation joints. Apart from the imperfect manufacturing and installation of the rotary encoder, the variations of ambient temperature could cause the angle measurement error of the rotary encoder. According to the characteristics of the 2π periodicity of the angle measurement at the stationary temperature and the complexity of the effects of ambient temperature changes, the method based on the Fourier expansion-back propagation (BP) neural network optimized by genetic algorithm (FE-GABPNN) is proposed to improve the angle measurement accuracy of the rotary encoder. The proposed method, which innovatively integrates the characteristics of Fourier expansion, the BP neural network and genetic algorithm, has good fitting performance. The rotary encoder that is installed in the rotation joint of the articulated coordinate measuring machine (ACMM) is calibrated by using an autocollimator and a regular optical polygon at ambient temperature ranging from 10 to 40 °C. The contrastive analysis is carried out. The experimental results show that the angle measurement errors decrease remarkably, from 110.2″ to 2.7″ after compensation. The mean root mean square error (RMSE) of the residual errors is 0.85″.

## 1. Introduction

The precision measuring instruments such as laser tracker, articulated coordinate measuring machine (ACMM) and total station are widely used in the area of field measurement for its portability. The rotation joint with high-accuracy angle sensor is the core component of these instruments. The angle measurement accuracy of angle sensors has great influence on the measurement performance of these precision instruments. Rotary encoders are widely used as high-accuracy angle sensors in these instruments. The ambient temperature in industrial site varies greatly from place to place. The measurement accuracy of these precision instruments is influenced greatly by ambient temperature. One main reason for this is that the angle measurement error induced by temperature will induce large measurement error of these instruments. Many methods have been developed to improve the angle measurement accuracy of the rotary encoder. The discrete angle measurement error values were obtained by the use of an autocollimator and a regular optical polygon to provide reference angle [1,2,3,4,5]. The eccentricity of the grating disk accounts for the majority of the angle measurement error. An eccentricity error model was built, and angle measurement accuracy was improved by compensating the eccentricity error [1,2,3]. Gao et al. analyzed the discrete values by fast Fourier transform (FFT) and calculated the constants with particle swarm optimization (PSO) to solve the problem of the non-convergence of the traditional least square method [4]. Hong et al. set up the compensation model which is based on the radial basis function (RBF) neural network [5]. Geckeler et al. built an experimental set-up that contains two rotary tables, an autocollimator and a five-sided calibration polygon. The Fourier methods involving transfer functions were presented to calibrate the angle encoders. Different weighting schemes were tested to reduce the measurement uncertainty [6]. Deng et al. introduced a useful device composed of a high-precision angular sensor to calibrate the optical encoder. An error compensation model was built based on the Fourier expansion. The weights were optimized by using the adaptive different evolution (ADE) method for the advantages of high accuracy and robustness to initial weights [7]. Zhao et al. proposed a method named extreme learning machine-Fourier neural network (ELM-FNN) to correct the angle measurement error of the optical encoder. The weights of FNN were calculated by ELM, which has the advantage of higher accuracy [8].

Li et al. proposed a method of angular positioning error analysis of the rotary stage based on the Abbe principle without using an autocollimator or a regular optical polygon to reduce the error [9]. The Abbe principle was extended to the angular measurement area to improve the angle measurement accuracy [10]. The relationship between the angle measurement error and the eccentricity of grating disk error motions of rotating shaft was analyzed, and the error models were built. The angle measurement error was decreased significantly [11,12,13]. The multi-reading heads were set in an irregularly distributed way and the transfer function was used to realize self-calibration of rotary encoder installed in the precise rotary table [14,15]. The researches mentioned above proposed many methods to correct the angle measurement error of the rotary encoder effectively without considering the effect of the ambient temperature.

Considering the temperature effect of the rotary encoder, Yu et al. proposed a method based on Fourier expansion-polynomial fitting (FEPF) to improve the angle measurement accuracy [16]. The setting of the degree of polynomial was not done properly and the data analysis of repeated experiments was insufficient. Except this article, previous researches seldom took into consideration on the temperature effect of the angle measurement accuracy of the rotary encoder. Many relevant studies have been presented to compensate the temperature effect of sensors. Hu et al. proposed an incremental optical linear encoder error model and estimation method. The errors of the different positions of linear encoder were compensated by using the empirical mode decomposition and the linear least square fitting method. The errors of the different temperatures were compensated by the cross-correlation methods [17]. Liu et al. proposed a high-order nonlinear fitting method with the quadratic model evolution to reduce the temperature effects on the measurement accuracy of accelerometer [18]. Wang et al. presented a multiple regression model considering the temperature variables to improve the navigation accuracy of a single-axis rotational inertial navigation system [19]. Zhang et al. proposed a Fourier expansion-back propagation (BP) neural work to compensate the effect of the temperature of elasto-magneto-electric sensors. A two-dimensional polynomial fitting method was also proposed for comparison. It was concluded that the BP neural work method was more effective and robust [20]. Araghi et al. proposed a temperature-dependent model using the RBF neural network to compensate measurement errors of the micro-electromechanical systems (MEMSs) inertial sensors. The RBF neural network reached better compensation effect by handling nonlinearity inherent in sensors [21]. Xu et al. presented a temperature compensation method to improve the performance of the MEMSs accelerometer based on the improved BP neural network, with advantages of fast convergence speed and good fitting performance [22]. The neural network was widely used in measurement compensation of sensors. The genetic algorithm has the advantage in global search to avoid the network getting stuck with local minimum [23,24,25,26].

The structure of the article is introduced as follows. Section 2 presents the structure of the rotation joints. Section 3 describes the Fourier expansion-back propagation neural network optimized by genetic algorithm (FE-GABPNN). Section 4 presents the experimental system and analyzes the experimental results. The discussion and conclusions are presented in Section 5 and Section 6 separately.

The main contributions of this work are: (1) Through the experiments, it is found that the variations of ambient temperature could cause the significant angle measurement error of the rotary encoder installed in the ACMM; (2) a novel method based on the FE-GABPNN is proposed to compensate the angle measurement error of the rotary encoder. The Fourier expansion which is from 0 to 11 orders is used to build the error compensation model at the temperature of 20 °C without residual errors based on the character of periodicity of angle measurement errors. The improved BP neural network whose initial parameters are optimized by the genetic algorithm is used to compensate the angle measurement errors caused by ambient temperature ranging from 10 to 40 °C. The proposed method, which innovatively integrates the characteristics of Fourier expansion, BP neural network and genetic algorithm, especially has good performance in compensating the angle measurement errors of rotary encoders when the ambient temperature changes; (3) the method mentioned above has the potential of being applied to other precisions instruments which contain rotation joints, such as a laser tracker.

## 2. Rotary Encoder Installed in the Rotation Joint of ACMM

**Figure 1:**
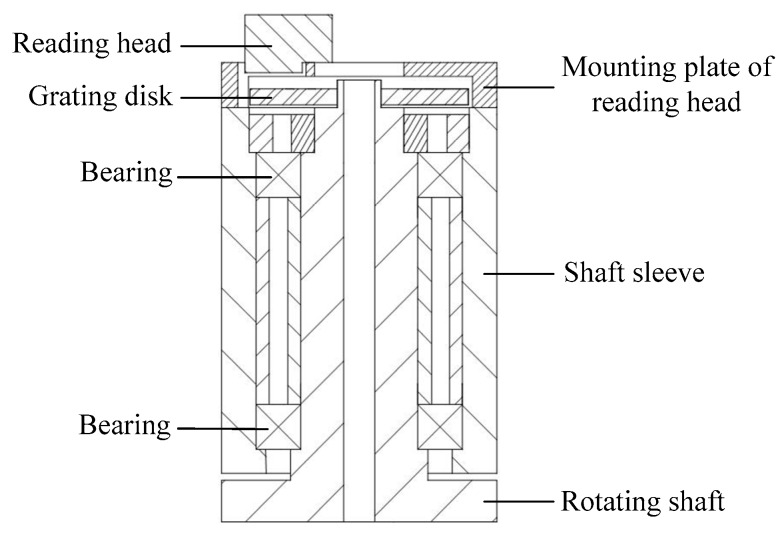
Diagram of the rotation joint.

ACMM is a kind of precision measuring instrument in tandem structure and it contains six rotation joints installed with rotary encoders. The structure of the rotation joint is shown in Figure 1.

The reading head is installed on the mounting plate of the reading head that is installed on the shaft sleeve, the grating disk is installed on the rotating shaft. The reading head is stationary and the grating disk installed on the rotating shaft is rotated relative to the reading head. The rotary encoder is composed of the reading head and the grating disk. The model of the rotary encoder is the Mercury 3000 (Celera MOTION Company, Bedford, MA, USA, resolution is 0.39″, measuring range is 360°, measurement accuracy is ±2.1″ before calibration, output is A-quad-B and digital index window). The angle measurement errors of the rotary encoder are mainly caused by error motions of the rotating shaft, manufacturing and installation eccentricity of the grating disk, and variations of ambient temperature.

## 3. Fourier Expansion-BP Neural Network Optimized by Genetic Algorithm

**Figure 2:**
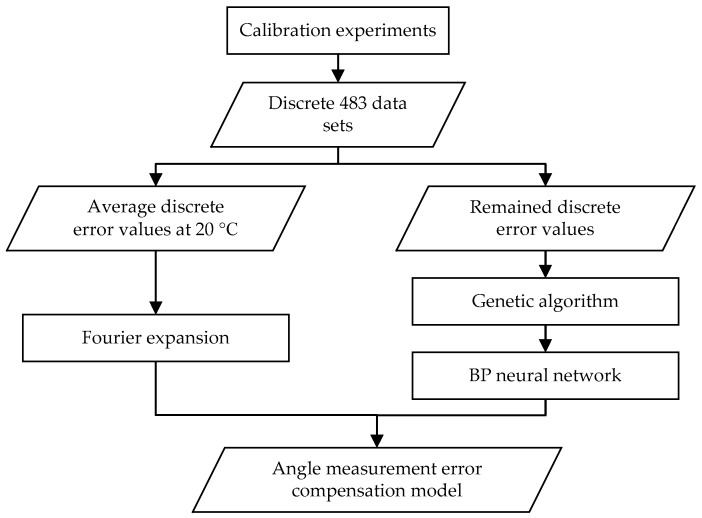
Structure of the Fourier expansion-back propagation (BP) neural network optimized by genetic algorithm (FE-GABPNN).

The angle measurement error compensation model was established by FE-GABPNN method. The discrete angle measurement error values were calibrated by using an autocollimator and a regular optical polygon with 23 faces at temperatures of 10, 15, 20, 25, 30, 35 and 40 °C separately. The repeated experiments were carried out three times at each temperature among the seven temperatures mentioned above. A total of (23×7×3=)483 data sets were obtained. Each data set included the ambient temperature T, the angular position of the rotary encoder *θ* and the relative discrete angle measurement error value Δθ. The structure of the FE-GABPNN method is shown Figure 2. Based on the characteristics of angle measurement errors of the rotary encoders, the proposed method innovatively integrated the characteristics of Fourier expansion, BP neural network and genetic algorithm to further improve the angle measurement accuracy.

In the following subsections, Section 3.1 provides the details of the Fourier expansion, Section 3.2 introduces the details of the BP neural network and Section 3.3 introduces the details of the genetic algorithm.

### 3.1. Fourier Expansion

The characters of angle measurement errors are circle closure and periodicity [5]. Fourier expansion method is simple with good fitting accuracy when the ambient temperature is not changed. The average values of angle measurement errors calibrated at temperature of 20 °C were set as the basic angle measurement errors. The angle measurement error compensation model at the temperature of 20 °C was built by the Fourier expansion, as shown in Equation (1):

(1) $$\varepsilon {\left(\theta \right)}^{20}=a_{0}+\sum_{i=1}^{11}\left(a_{i}\sin(i\theta )+b_{i}\cos(i\theta )\right).$$

The 23 discrete values were obtained in the calibration process, so the Fourier expansion which is from 0 to 11 orders can be used to fit the discrete angle measurement values without residual errors. The parameters $a_{0},a_{i},b_{i},i=1,\cdots ,11$ were calculated based on the least-squares method by using the average discrete values $\Delta {\bar{\theta }}_{k}^{20}\cdot k=1,2,\cdots ,23$. The algorithm details of the least-squares method are introduced in Appendix A.

### 3.2. BP Neural Network

The improved BP neural network optimized by the genetic algorithm was used to compensate the angle measurement errors caused by the ambient temperature. The BP neural network is illustrated in the following section. The remained discrete angle measurement error values $\Delta {\theta^{\prime }}_{m},m=1,2,\cdots ,483$ were obtained by subtracting the average discrete values $\Delta {\bar{\theta }}_{k}^{20}$ calibrated at temperature of 20 °C from the discrete angle measurement error values $\Delta \theta_{m}$ mentioned above.

#### 3.2.1. Structure of the BP Neural Network

**Figure 3:**
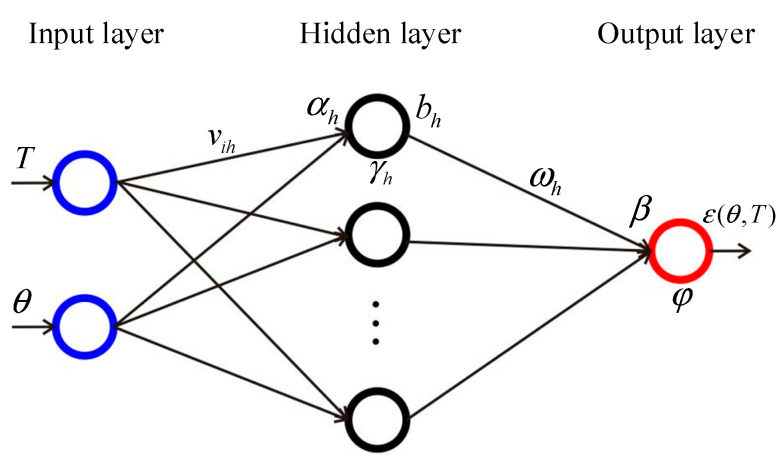
Structure of the BP neural network.

The structure of the BP neural network is shown in Figure 3. The BP neural network consists of one input layer, one hidden layer and one output layer. The remained discrete angle measurement error values were relative to the angular position and ambient temperature. So the input layer included two nodes, and the input variables were the angular position of rotary encoder *θ* and ambient temperature T. The output layer had one node and the predicted value was the compensation value of the angle measurement error ε(θ,T). There is no exact algorithm to determine the number of nodes of the hidden layer n [27]. Some empirical formulas are proposed to calculate the general number nodes of the hidden layer [28]. When the number of hidden layer nodes is small, the structure of the BP neural network is too simple to fit the discrete angle measurement error values properly. On the other hand, when there are too many nodes in the hidden layer, the BP neural network has the risk of overfitting. The number of hidden layer nodes is decided by the training results of the BP neural network, which is evaluated by the RMSE of the residual errors after compensation.

Further, $v_{ih}$ was the weight between the input layer and hidden layer; $\omega_{h}$ was the weight between the hidden layer and output layer; $\gamma_{h}$ was the bias of the hidden layer; φ was the bias of the output layer. The input value of the neural cell of the hidden layer was calculated by Equation (2):

(2) $$\alpha_{h}=v_{1h}T+v_{2h}\theta .$$

The output value of the node of the hidden layer was $b_{h}=f(\alpha_{h}-\gamma_{h})$. The input value of the node of the output layer was calculated by Equation (3):

(3) $$\beta =\sum_{h=1}^{n}\omega_{h}b_{h}$$

The output value of the node of the output layer was ε(θ,T)=g(β−φ). Here, i=1,2;h=1,⋯,n. The transfer functions we used were shown as follows.

(4) $$b_{h}=\frac{2}{1+e^{-2(\alpha_{h}-\gamma_{h})}}-1.$$

(5) ε(θ,T)=β−φ.

#### 3.2.2. Training of the BP Neural Network

**Figure 4:**
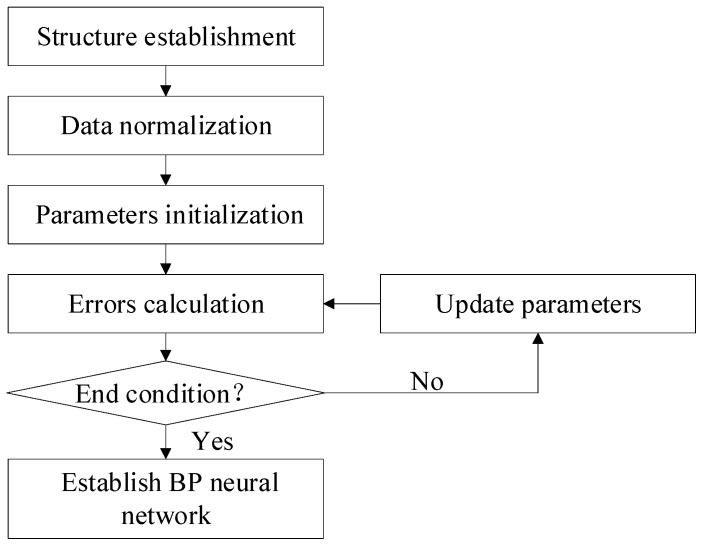
Flowchart of the BP neural network.

The flowchart of the BP neural network is shown in Figure 4.

*Structure establishment*. The structure of the BP neural network is described in detail in Section 3.2.1. There were 483 data sets obtained from the above-mentioned calibration process, and the data sets are randomly divided, with 90% used for training, 10% for validation.

*Data normalization*. All the data sets, which consist of the input values and expected values, were normalized within [0, 1]. The normalization process was shown in Equation (6).

(6) $$T_{m}=\frac{T_{m}-T_{m\min}}{T_{m\max}-T_{m\min}},\theta_{m}=\frac{\theta_{m}-\theta_{m\min}}{\theta_{m\max}-\theta_{m\min}},\Delta \theta_{m}^{\prime }=\frac{\Delta \theta_{m}^{\prime }-\Delta \theta_{m\min}^{\prime }}{\Delta \theta_{m\max}^{\prime }-\Delta \theta_{m\min}^{\prime }},m=1,2,\cdots ,483.$$

$T_{m\max},\theta_{m\max},\Delta \theta_{m\max}^{\prime }$ were the maximum values of the ambient temperature, angular position and remained discrete angle measurement error values, separately, and $T_{m\min},\theta_{m\min},\Delta \theta_{m\min}^{\prime }$ were the minimum values.

*Parameters initialization.* Then the initialized parameters, which consist of the weights $v_{ih}$, $\omega_{h}$ and biases $\gamma_{h}$, φ, are set to random numbers within [−1, 1].

*Errors calculation.* The predicted values $\varepsilon {\left(\theta ,T\right)}_{m}$, which are the compensation values of the angle measurement error in this situation, were calculated by Equations (2)–(5). Then the residual errors were calculated by subtracting the predicted values $\varepsilon {\left(\theta ,T\right)}_{m}$ from the expected values $\Delta \theta_{m}^{\prime }$.

*Update parameters.* When mean squared error (MSE) of the residual errors is not less than the target training error, or iterations are not completed, the weights and biases are updated through the corresponding algorithms. The residual errors are back-propagated through the neural network, the Levenberg–Marquardt (LM) algorithm was used to adjust the weights and biases, so as to build the proper compensation model and reduce the residual errors. The LM algorithm is more efficient for training the moderate-sized neural networks, which is up to several hundred weights, with good prediction performance [20,29].

The BP neural network is built when it meets the end condition.

### 3.3. Genetic Algorithm

**Figure 5:**
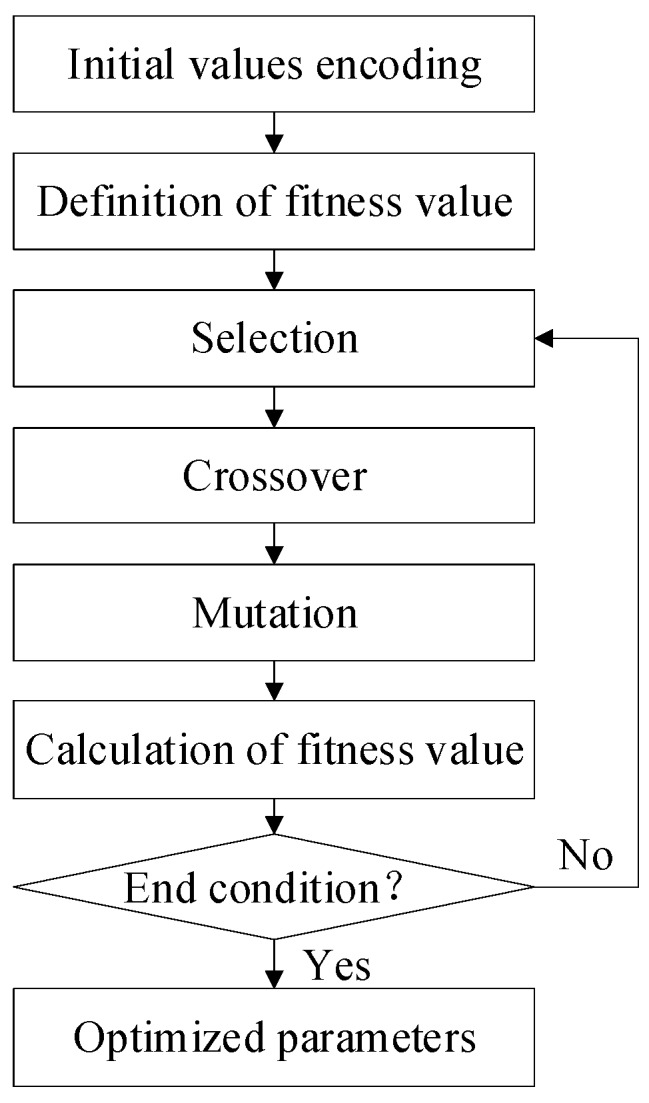
Flowchart of the genetic algorithm.

The prediction performance of the BP neural network is influenced by the initial weights and biases. The genetic algorithm is used to optimize the initial weights and biases to improve the prediction performance. The flowchart of the genetic algorithm is presented in Figure 5.

*Initial values encoding.* A population consists of different individuals. Each one is a string of the real numbers which are consisted of the weights between the input layer and hidden layer $v_{ih}$, the biases of the hidden layer $\gamma_{h}$, the weights between the hidden layer and output layer $\omega_{h}$, and the bias of the output layer φ. The parameters mentioned above are encoded in order.

*Definition of fitness value.* The MSE of the residual errors is set as the fitness value of individual F in genetic algorithm, as shown in Equation (7):

(7) $$F=\frac{1}{483}\sum_{m=1}^{483}{\left(\Delta \theta_{m}{}^{\prime }-\varepsilon {\left(\theta ,T\right)}_{m}\right)}^{2}.$$

*Selection.* The selection algorithm used in this article is the roulette method based on the proportionate fitness. The selection probability is calculated by Equation (8):

(8) $$\begin{array}{c} f_{n}=1/F_{n}\\ p_{n}=\frac{f_{n}}{\sum_{i=1}^{N}f_{n}} \end{array}.$$

Here, $F_{n}$ is the fitness value of individual n, $p_{n}$ is the selection probability of individual n and N is the size of individuals. The smaller the fitness value of individual is, the higher the selection probability will be. The individuals which are selected will be conducted in the subsequent operations.

*Crossover.* The same parameters of two different individuals are changed by using the crossover algorithm, as shown in Equation (9):

(9) $$\left\{ \begin{array}{l} {a_{mk}}^{\prime }=a_{mk}(1-w)+a_{nk}w\\ {a_{nk}}^{\prime }=a_{nk}(1-w)+a_{mk}w \end{array}\right.,$$

where $a_{mk}$, $a_{nk}$ are the values of the parameter numbered k of the individual m and n, separately. The parameter numbered k is selected randomly from the whole weights and biases, individuals m, n are selected randomly from the population. w is a random value within [0, 1].

*Mutation.* The mutation algorithm is shown in Equation (10):

(10) $${a_{ij}}^{\prime }=\left\{ \begin{array}{l} a_{ij}+\left(a_{ij}-a_{\max}\right)*f\left(e\right)r>0.5\\ a_{ij}+\left(a_{\min}-a_{ij}\right)*f\left(e\right)r\leq 0.5 \end{array}\right..$$

Here $a_{ij}$ is the value of the parameter numbered j of the individual i. $a_{\max}$, $a_{\min}$ are the maximum and minimum of the parameter numbered j of the whole individuals, separately. $f\left(e\right)=r_{e}{\left(1-e/E_{\max}\right)}^{2}$, where $r_{e}$ is a random value within [0, 1]. e is the current evolution number. $E_{\max}$ is the maximum evolution value. r is a random value within [0, 1].

*Calculation of fitness value.* The selection, crossover and mutation mentioned above are operated N times separately. Then the fitness value of each individual is calculated, the individual whose fitness value is the smallest is obtained and then the corresponding parameter values are obtained.

*Optimized parameters.* One evolution process is consisted of the processes mentioned above. The whole individuals of population evolve $G_{\max}$ times. At last, the individual whose fitness value is the smallest in the whole evolutions is found, the corresponding optimized weights and biases are obtained and used as the initial parameters of the BP neural network.

## 4. Experimental Results

### 4.1. Calibration Experiment

**Figure 6:**
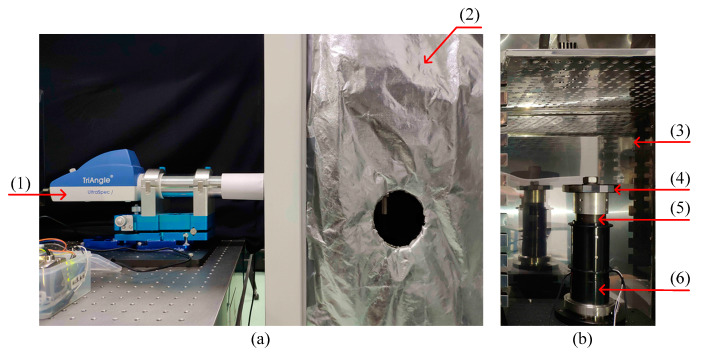
(**a**) Experimental system (the door of thermotank is not shown in Figure 6). (**b**) Internal view of thermotank. The experimental system is composed of (1) autocollimator, (2) thermal barrier (3) thermotank, (4) regular optical polygon, (5) rotation joint and (6) foundation support.

The experimental system is shown in Figure 6. The autocollimator and regular optical polygon were used to obtain the discrete angle measurement error values of the rotary encoder [30]. The model of autocollimator was TriAngle US 300-57 (TRIOPTICS Company, Wedel, Germany, with resolution of 0.005″ and measurement accuracy of 0.25″). The rotation joint which was installed close to the bottom of the ACMM is shown as an example. The structure of the rotation joint is shown in Figure 1. The temperature sensor was fixed on the mounting plate of the reading head. The temperature sensor used in the experiment was DS18B20 (Maxim, San Jose, CA, USA, with resolution of 0.06 °C and accuracy of 0.5 °C). The rotation joint was installed on the foundation support which the shaft sleeve is fixed on. The installation situation of the rotation joint in experiment and industrial field were same. The foundation support with the rotation joint was placed in the thermotank. The regular optical polygon with 23 faces and rotating shaft were fixed by the use of a clamp. The angle measurement error of the autocollimator will not be caused by the small eccentric installation of the regular optical polygon. The autocollimator and foundation support were adjusted to make sure that the light beam was perpendicular to the regular optical polygon. The position of the regular optical polygon was adjusted relative to the clamp to make sure that the indicating value of the rotary encoder θ was less than 0.2° when the autocollimator was aimed at the first working face of the regular optical polygon. The initial position error is negligible, because 0.2° is small compared with 360°. Then the regular optical polygon was fixed by fastening the nut.

Set the temperature in the thermotank and maintained the temperature for a long enough time to make sure the internal and external parts of the ACMM reach the same temperature. The thermal barrier was used to make sure that the variation of temperature was less than 0.2 °C during the calibration. The rotating shaft was rotated by hand without using motor for the reason that it was similar to the practical application of ACMM. The indicating value of the rotary encoder $\theta_{k}$ and the indicating value of the autocollimator along the horizontal direction $\delta_{Xk},k=1,\cdots ,23$ were recorded simultaneously, when the autocollimator was aimed at each working face of the regular optical polygon. The discrete angle measurement error values $\Delta \theta_{k}^{T}$ at current ambient temperature T were calculated by Equation (11):

(11) $$\Delta \theta_{k}^{T}=(\theta_{k}-\delta_{Xk})-(\theta_{1}-\delta_{X1})-360/23*(k-1).$$

All the calibration data mentioned in the previous chapter were obtained in the same way.

During the experiments, it took within 30 min to install and adjust the devices. It usually took 4 h to change and maintain the temperature in the thermotank to make sure the different parts of ACMM reach the same set temperature at each specific temperature. It took within 20 min to finish the calibration process, which consists of repeated experiments carried out three times at each specific temperature. It took about 40 min to train all the calibration data and build the error compensation model when the time of repeated trials of the BP neural network is included.

### 4.2. Compensation Results

**Figure 7:**
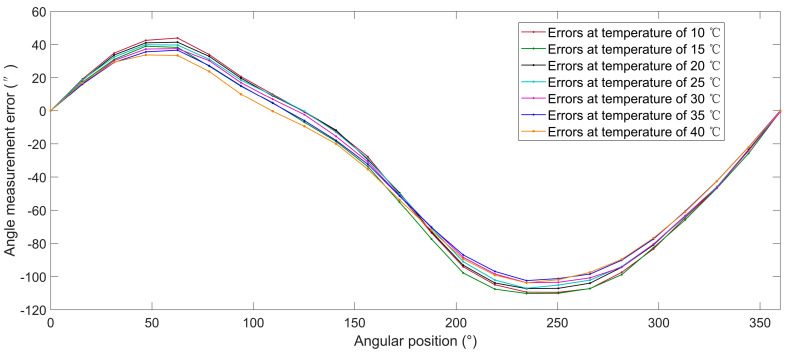
Average angle measurement error before calibration.

The average discrete angle measurement error values before compensation at temperatures of 10, 15, 20, 25, 30, 35 and 40 °C are shown in Figure 7. The angle measurement errors ranged from −110.2″ to 43.9″. The angle measurement errors caused by the variation of temperature at the same angular position reached up to (−9.7″, 7.0″).

**Figure 8:**
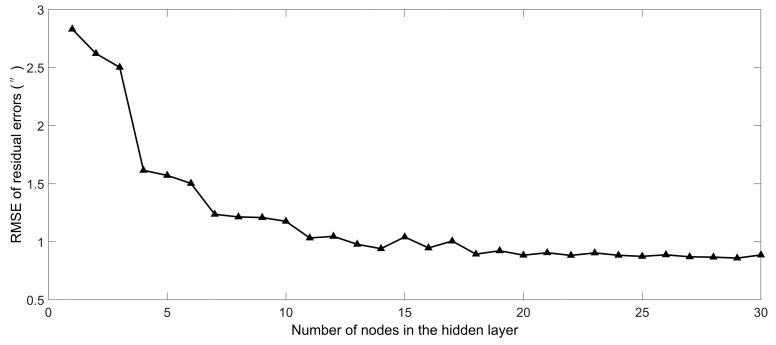
Performance of the BP neural network whose numbers of nodes in the hidden layer is different.

The angle measurement errors at the temperature of 20 °C were compensated by using the Fourier expansion mentioned above. The remained angle measurement errors were compensated by the improved BP neural network optimized by the genetic algorithm. It was obvious that 20 nodes in the hidden layer was optimal from Figure 8. Here a 2-20-1 BP neural network was selected.

**Figure 9:**
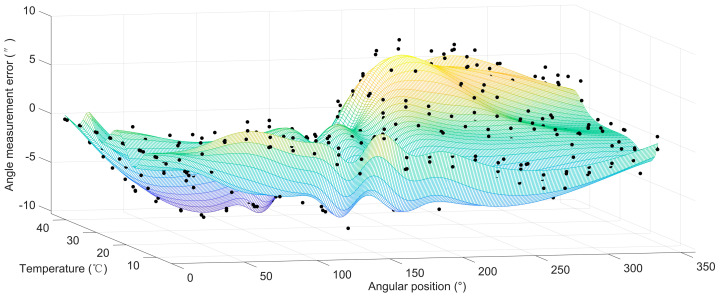
Compensation effect of the Fourier expansion-BP neural network optimized by genetic algorithm (FE-GABPNN) method.

According to the convergence condition of the fitness value of individuals in genetic algorithms, the total number of individuals N was set to 30, the maximum evolution number $G_{\max}$ was set to 25. The compensation effect of the FE-GABPNN is presented in Figure 9. The RMSE of the residual errors after compensation was 0.84″, the residual errors were within (−2.2″, 2.5″).

### 4.3. Contrastive Analysis

#### 4.3.1. Two-Dimensional Polynomial Fitting

The two-dimensional polynomial fitting is widely used to fit the model that has two independent variables and one dependent variable. The mathematical model of the two-dimensional polynomial fitting method is expressed by Equation (12):

(12) $$\begin{array}{l} \varepsilon (\theta ,T)=p_{00}+p_{10}\theta +p_{01}T+p_{20}\theta^{2}+p_{11}\theta T+p_{02}T^{2}+\ldots \\ +p_{50}\theta^{5}+p_{41}\theta^{4}T+p_{32}\theta^{3}T^{2}+p_{23}\theta^{2}T^{3}+p_{14}\theta T^{4}+p_{05}T^{5} \end{array}$$

**Figure 10:**
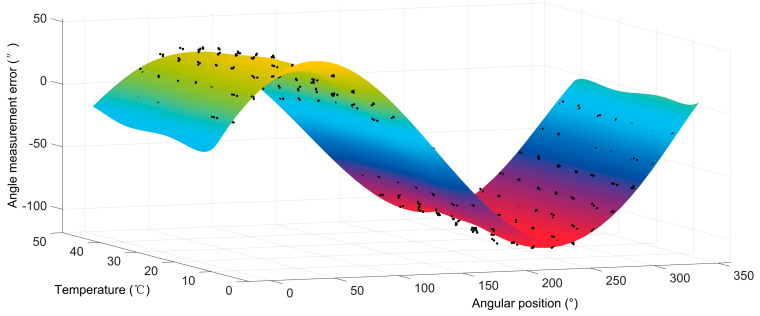
Compensation effect of the two-dimensional polynomial fitting method.

Using the above-mentioned 483 data sets, the parameters $p_{00},p_{10},\cdots ,p_{05}$ are obtained by the linear least square fitting method. The compensation effect of the two-dimensional polynomial fitting method is presented in Figure 10. The RMSE of the residual errors after compensation is 3.69″. The extreme deviation of the residual errors after compensation is (−9.1″, 10.6″).

#### 4.3.2. FEPF Method

The FEPF method is proposed to improve the angle measurement accuracy of rotary encoders [16]. First, the compensation model of angle measurement error at each specific temperature is built by using Fourier expansion, as shown in Equation (13). Then the relationship between the coefficients of the Fourier series and ambient temperature is established by using of polynomial fitting separately, as shown in Equation (14). The orders of Fourier series are from 0 to 7, the degree of polynomial is 5.

(13) $$\varepsilon (\theta ,T)=f_{a_{0}}\left(T\right)+\sum_{i=1}^{7}\left(f_{a_{i}}\left(T\right)\sin\left(i\theta \right)+f_{b_{i}}\left(T\right)\cos\left(i\theta \right)\right).$$

(14) $$\{\begin{array}{c} f_{a_{0}}\left(T\right)=p_{a_{0}1}T^{5}+p_{a_{0}2}T^{4}+\cdots +p_{a_{0}5}T+p_{a_{0}6}\\ f_{a_{i}}\left(T\right)=p_{a_{i}1}T^{5}+p_{a_{i}2}T^{4}+\cdots +p_{a_{i}5}T+p_{a_{i}6}\\ f_{b_{i}}\left(T\right)=p_{b_{i}1}T^{5}+p_{b_{i}2}T^{4}+\cdots +p_{b_{i}5}T+p_{b_{i}6} \end{array}.$$

Using the above-mentioned 483 data sets, the parameters $p_{a_{0}1},\cdots ,p_{a_{0}6},p_{a_{i}1},\cdots ,p_{b_{i}6},i=1,2,\cdots ,7$ are calculated by the least square fitting method. The residual errors are calculated by subtracting the compensation values from the original errors. The RMSE of the residual errors after compensation is 1.20″. The extreme deviation of the residual errors after compensation is (−2.1″, 2.9″).

#### 4.3.3. Compensation Effect Comparison

The Fourier expansion-standard BP neural network (FE-BPNN) method, without using genetic algorithm, and the standard BP neural network (BPNN) method, without using Fourier expansion or genetic algorithm, are presented for comparison. The 25 nodes in the hidden, which is expected to have the best performance, are set when using the BPNN method. The same experiments’ data sets are used as the training and test data.

Each approach is evaluated using the mean of the RMSE and the extreme deviation of the residual errors after compensation. The unit of the RMSE of the residual errors and the angle measurement errors are the same. Using the RMSE has the advantage of reflecting the magnitude of the residual errors directly compared with the MSE.

For the FE-GABPNN, FE-BPNN and BPNN methods, the repeated trials are carried out 20 times. The results are presented in Table 1. The compensation results of two-dimensional polynomial fitting and FEPF are presented in Table 2.

Compared with the FE-BPNN and BPNN methods, the FE-GABPNN method shows a better compensation effect by evaluating the mean RMSE and mean extreme deviation of residual errors. Compared with the FEPF method proposed in a recent study, the extreme deviation of the residual errors by using FE-GABPNN method is almost the same, but the RMSE of the residual errors decreases from 1.20″ to 0.85″.

## 5. Discussion

The angle measurement accuracy of the rotary encoder installed in the rotation joint is mainly influenced by error motions of rotating shaft, installation eccentricity, imperfect manufacturing of the grating disk and variations of ambient temperature. In the article, a method based on FE-GABPNN is proposed to compensate the angle measurement error of the rotary encoder. The Fourier expansion method has the advantage of compensating periodic errors and the improved BP neural network optimized by the genetic algorithm method has the advantages of nonlinear fitting capability, regardless of the exact error model of the angle sensors and overcoming the local minimum. The FE-GABPNN method innovatively integrates the characteristics of Fourier expansion, BP neural network and genetic algorithm.

The rotary encoder installed in the rotation joint of ACMM is calibrated by the experiment. The experimental results show that over the ambient temperature range of 10 to 40 °C, the FE-GABPNN is superior to the FE-BPNN, BPNN, two-dimensional polynomial fitting and FEPF. The FE-GABPNN, which further decreases the RMSE of the residual errors, has better performance in compensating the angle measurement errors of rotary encoders when the ambient temperature changes. The FE-GABPNN method mentioned above has the potential of being applied to other precision instruments which contain rotation joints, such as a laser tracker.

By using the FE-GABNN proposed in this article, the residual errors after compensation is almost the same as the random errors of the angle measurement errors. It is hard to improve the angle measurement accuracy of rotary encoders based on the current devices when using single reading head. From our previous research work carried out at the stationary temperature, the random errors are decreased in a certain extent by using the double reading heads which are set in a regularly distributed way. The reason for this is that some error sources of random errors have the opposite effects on the double reading heads. The random errors caused by the variations of ambient temperature may have the similar characteristics. In addition, according to the researches [14,15], different arrangements of multi-reading heads have different compensation effect.

So, in future work, installing multi-reading heads is considered as the effective way to improve the angle measurement accuracy of the rotary encoders when the ambient temperature effect is considered, and the algorithm based on the multi-reading heads will be researched.

## 6. Conclusions

In this paper, a novel method based on FE-GABPNN is proposed to improve the angle measurement accuracy of the rotary encoders installed in the rotation joints of ACMM. The angle measurement errors decrease remarkably, from 110.2″ to 2.7″ after compensation. The mean RMSE of residual errors after compensation is 0.85″. Installing multi-reading heads in a proper way and developing the related algorithm is the next step to further improve the angle measurement accuracy of the rotary encoders.

## Figures and Tables

**Table 1:** Comparative result by Fourier expansion-BP neural network optimized by genetic algorithm (FE-GABPNN), Fourier expansion-standard BP neural network (FE-BPNN) and standard BP neural network (BPNN).

Item	Mean RMSE (″)	Mean Extreme Deviation (″)
FE-GABPNN	0.85	−2.3, 2.7
FE-BPNN	0.90	−2.6, 2.8
BPNN	1.38	−4.1, 4.1

**Table 2:** Compensation results of two-dimensional polynomial fitting and Fourier expansion-polynomial fitting (FEPF).

Item	RMSE (″)	Extreme Deviation (″)
Two-Dimensional Polynomial Fitting	3.69	−9.1, 10.6
FEPF	1.20	−2.1, 2.9

## Appendix A

This appendix introduces the algorithm details of the least-square method to calculate the parameters $a_{0},a_{i},b_{i},i=1,\cdots ,11$ mentioned in Section 3.1.

The matrix M is defined as $M={\left[a_{0}\;a_{1}\;b_{1}\;a_{2}\;b_{2}\cdots a_{11}\;b_{11}\right]}^{T}$.

The matrix $E_{mea}$ is defined as $E_{mea}={\left[\Delta {\bar{\theta }}_{1}^{20}\;\Delta {\bar{\theta }}_{2}^{20}\;\Delta {\bar{\theta }}_{3}^{20}\cdots \Delta {\bar{\theta }}_{22}^{20}\;\Delta {\bar{\theta }}_{23}^{20}\right]}^{T}$.

The matrix A is defined as

$A=\left[\begin{array}{cccccccc} 1 & \sin\theta_{1} & \cos\theta_{1} & \sin2\theta_{1} & \cos2\theta_{1} & \cdots & \sin11\theta_{1} & \cos11\theta_{1}\\ 1 & \sin\theta_{2} & \cos\theta_{2} & \sin2\theta_{2} & \cos2\theta_{2} & \cdots & \sin11\theta_{2} & \cos11\theta_{2}\\ 1 & \sin\theta_{3} & \cos\theta_{3} & \sin2\theta_{3} & \cos2\theta_{3} & \cdots & \sin11\theta_{3} & \cos11\theta_{3}\\ \vdots & \vdots & \vdots & \vdots & \vdots & & \vdots & \vdots \\ 1 & \sin\theta_{22} & \cos\theta_{22} & \sin2\theta_{22} & \cos2\theta_{22} & \cdots & \sin11\theta_{22} & \cos11\theta_{22}\\ 1 & \sin\theta_{23} & \cos\theta_{23} & \sin2\theta_{23} & \cos2\theta_{23} & \cdots & \sin11\theta_{23} & \cos11\theta_{23} \end{array}\right]$. Where $\theta_{i},i=1,2,\cdots ,23$ is the angular position of rotary encoder.

The matrix M is calculated by the following equation: $M={\left(A^{T}A\right)}^{-1}A^{T}E_{mea}$. The parameters $a_{0},a_{i},b_{i},i=1,\cdot \cdot \cdot ,11$ are calculated.
